# Differences in opinions of occupational physicians on the required competencies by field of practice: results of an international Delphi study

**DOI:** 10.1186/s12909-018-1139-9

**Published:** 2018-04-02

**Authors:** Evangelia Demou, Drushca Lalloo, Ewan B. Macdonald

**Affiliations:** 1 MRC/CSO Social and Public Health Sciences Unit, Institute of Health and Wellbeing, University of Glasgow, Glasgow, G2 3QB UK; 2 Healthy Working Lives Group, Institute of Health and Wellbeing, University of Glasgow, Glasgow, G12 8RZ UK

**Keywords:** Occupational physician, Occupational health, Competency, Clinician, Manager, Academic, Training

## Abstract

**Background:**

The activities and work demands of medical professionals, including occupational physicians (OPs), fall into three categories: clinical, academic, and administrative. Work demands of an OP consist of these three categories and additional specialty specific roles and competencies. Research on the core competencies and skills required for OPs have identified high levels of consensus amongst OPs internationally, however these opinions have not been examined between areas of practice specific groups. Furthermore, it has been identified that to a large extent academics are often the group who define the skills required of OPs. The aim of this study is to compare the opinions of OPs grouped by field of practice on the common core competencies required for occupational health (OH) practice using results from an international survey.

**Methods:**

An international modified Delphi study conducted among OPs, completed in two rounds (Rating-Round 1; Ranking-Round 2) using developed questionnaires based on the specialist training syllabus of a number of countries and expert discussions. Respondents were categorised as Physician, Manager/Physician, and Academic/Physician, based on self-reported job titles and place of work.

**Results:**

There was good agreement between the Physician and Manager/Physician groups, with the Academic/Physician group deviating the most. The top three and bottom three principle domains (PDs) were in good agreement across all groups. The top three were clinically based and would be considered core OH activities. The PDs with considerable intergroup variance were Environmental Issues Related to Work Practice and Communication Skills, categories which may reflect direct relevance and relative importance to the job tasks of respective groups.

**Conclusion:**

This study demonstrated general agreement between the three occupational groups. Academic/Physician opinions deviate the most, while good agreement is depicted between the Physician and Manager/Physician groups. The findings of this study can help identify potential gaps in training requirements for OPs and be used as a stepping stone to developing training programmes that are reflective of practice and tailored for those predominantly undertaking these specific roles.

**Electronic supplementary material:**

The online version of this article (10.1186/s12909-018-1139-9) contains supplementary material, which is available to authorized users.

## Background

The range of activities required of medical professionals is well recognised. Previous research has shown that professional work demands of physicians fall into three categories: clinical, academic, and administrative [1] and this diverse nature of work has been identified as an important driver in the selection of one’s medical specialty [2]. Occupational Health (OH) practice is no exception. Work demands of an Occupational Physician (OP) consist of these three recognised categories and additional specialty specific roles and competencies, including for example assessment of workplace hazards, knowledge of health & safety and disability legislation, and environmental assessments, [3–9]. Research studies on OP competencies have been undertaken internationally [4, 6, 10]. Delclos et al. [4] demonstrated important differences in competencies and curricula between OH professional groups. In a European study, Macdonald et al. [6] demonstrated that advancements in OH practice were not aligned with the views of OPs on their training needs, which appeared to be focused on traditional core competencies. In a recent follow-up international study, it appears that consensus on common core competencies and priorities for OPs worldwide has not changed significantly, although it is acknowledged that the meaning and interpretation of what these competencies now encompass have evolved to accommodate the changing workplace and to include the concept of medical risk assessment [10]. A difference in opinion regarding competencies for OPs is recorded when examining the customer perspective, e.g. employers and employees [8]. Occupational Hazards to Health were consistently ranked as the most important competency by OPs [6, 10], whereas customers of OH services considered Law and Ethics the most important skill for OPs [8].

Research is an increasingly important aspect of higher medical training for many doctors [1, 11–14], both in terms of teaching and educational supervision as well as research in context-specific areas [13–15]. Academic medicine plays an important role and several initiatives exist that attempted to enhance the number of clinical academics, the quality of the conducted research, while safeguarding service provision [15–17]. Concurrently working in clinical practice and academia, offers opportunities of multidisciplinary and translational work from practise to theory and vice versa, and to influence industry, policy and practice [18]. Clinical academics are often also involved in medical and non-medical teaching [18]. A recent report by the Medical Schools Council reported that “Clinical academics are responsible for delivering the undergraduate curriculum, inspiring and educating the next generation of doctors, and they contribute substantially to postgraduate medical training” [18]. In the UK, it has been reported that approximately 5 % of medical consultants work as clinical academics [18].

The importance of good management skills is becoming increasingly apparent within health care [5]. It has been identified that the former has effects, not only on financial management, but on the quality of care provided [5]. Differences in management skills and management approaches may impact on quality of service in different ways, including possible effects on the stress and/or wellbeing of their staff which, in turn, is directly related to the quality of care produced [19].

Occupational medicine (OM) can contribute significantly to good management in healthy enterprises. The OPs role is to protect and promote the health and work ability of workers. If OPs are to make a maximum contribution to employees’ work ability and health and safety at work, they need to have the appropriate skills. OM is distinctive from other medical specialties in that it is in the main practised out with the National Health Service (NHS) in the UK and in industry. While previous studies have examined the professional development of OPs in terms of practice competencies and curricula, they have not examined intra-OP group differences, and specifically with regards to their job/predominant area of practice [4, 6, 10]. Furthermore, a report for the EEF (The Manufacturers’ Organisation) and the Health and Safety Executive reported that “it is still largely academics who define the skills of the occupational physicians who will be employed by industry” [8]. Given the evolution of OH practice, with increasing focus on service provision and reduction of emphasis on academic activities, we aim to compare the perspectives of occupational physicians’ on their required competencies by field of practice, using results from an international survey [10]. The specific focus of this paper is on the differences expressed between OPs undertaking predominantly clinical, predominantly managerial and predominantly academic roles, the three main categories within the scope of OH practice. The findings of this study can help identify differences in opinions and potential gaps in training requirements for OPs.

## Methods

### Delphi questionnaires

An international modified Delphi study was conducted among OPs in various countries around the world [10]. The study was completed in two rounds using developed questionnaires based on the specialist training syllabus of a number of countries, expert panel reviews and conference discussions. Details of the conducted Delphi study are described elsewhere [10]. The first round, was the ‘rating’ round and respondents were asked to indicate the relative importance of the included items [10]. The ‘rating’ questionnaire comprised of 12 principal domains covering the different topic areas of OH practice and within these were subsection items detailing specific competencies relating to that domain. The round 2 ‘ranking’ questionnaire retained the same 12 principal domains from the first round but included new subsection items suggested by round 1 respondents [10]. Respondents were asked to rank the principle domains and their subsection items. Both questionnaires were circulated to the same key contacts and they were asked to distribute the online questionnaire using a SMART survey link to their networks [10]. Specialist OPs that received the link were invited to participate irrespective of whether they had taken part in round 1 or not. Information on the study was embedded in the beginning of each electronic survey and participants were required to complete a consent question to proceed with survey completion.

Participants were asked to choose their area of practice (more than one option allowed) and to provide their job title. These were screened and respondents were categorised into three main job categories after assessment of the self-declared job titles and place of work provided by respondents. If respondents were solely involved in clinical OH practice, they were categorised as Physicians; if they had a management title, they were labelled Manager/Physicians and if they had an academic role the Academic/Physician category was applied. Areas of current OH practise comprised work in a healthcare setting, for example a hospital (healthcare), public/private sector organisations (industry), participation in teaching or research (academic) or any work sector not covered by these (other).

Ethics approval to undertake this study was provided by the University of Glasgow, College of Medical, Veterinary & Life Sciences Ethics Committee [200130150].

### Analysis

#### Round 1

Respondents were first asked whether OPs should be competent in the Principle domains, answering Yes, No, or Not Relevant. If they answered Yes, they were then asked to give each domain subsection item a separate score from 1 to 5 relating to the importance of the subject. A score of 1 indicated that the item was of least importance and 5 it was absolutely necessary. The data was analysed using SPSS Statistics 21 [20]. The 12 principal domains were treated separately. For each item in each of these subsections, the scores were averaged for all respondents and per job title grouping. Analyses were carried out to identify possible variations in responses between OPs identified as having different roles/titles. These three different groups comprised of Physicians; Manager/Physicians; Academic/Physician and Trainees. Comparisons of the relative importance to respondents of the 12 principal domains per job grouping were made with the Wilcoxon signed rank test.

#### Round 2

Responses to the second questionnaire were analysed by summing the rank orders to produce a mean score for each item within each of the 12 domains. As some sections had as many as 12 items and some as few as two the mean scores were standardised to a 1–10 scale, to allow comparison of the relative importance of items in different subsubsections [10]. Subsection mean standardised scores were subsequently weighted using a scale from 1 to 12 based on the ranking order of their respective principle domain [10].

## Results

### Demographics

In total 336 responses were received in round 1 of the Delphi survey and 232 in round 2 [10]. Removing missing values for job description, there were 332 and 232 respondents in Rounds 1 & 2, respectively for this analysis. In both Rounds 1 & 2 the majority of respondents were Physicians (69% (*n* = 228_ and 71% (165), respectively), followed by Manager/Physicians, (18% (*n* = 60) and 19% (*n* = 43), respectively), then Academic/Physicians (12% (*n* = 39) and 10% (*n* = 23), respectively), with the Trainee group contributing to less than 2% (n_Round1_ = 5; n_Round2_ = 1) of the responses. Respondent demographic details, area of practice, and years of experience by job group are demonstrated in Table 1. The sample size of the ‘Trainee’ group was insufficient to draw valid conclusions and was hereafter excluded from the analysis.

**Table 1:** Responses by age, sex, continents, job title and years of experience for Rounds 1 and 2

Job Title	Physician		Manager/Physician		Academic/Physician		Trainee	
	Round 1	Round 2	Round 1	Round 2	Round 1	Round 2	Round 1	Round 2
	n (%)	n (%)	n (%)	n (%)	n (%)	n (%)	n (%)	n (%)
Total	228 *(68.7)*	165 *(71.1)*	60 *(18.1)*	43 (*18.5*)	39 *(11.7)*	23 *(9.9)*	5 *(1.5%)*	1
Age range								
25–34	8 *(3.0)*	3 *(2.0)*	1 *(2)*	1 *(2)*	0 *(0)*	0 (0)	1 *(20)*	0 *(0)*
35–44	43 *(19)*	39 *(24)*	8 *(13)*	7 *(16)*	1 *(3)*	1 (5)	3 *(60)*	0 *(0)*
45–54	79 *(35)*	50 *(30)*	20 *(33)*	12 *(28)*	15 *(38)*	9 (41)	1 *(20)*	1 *(100)*
55–64	72 *(32)*	59 *(36)*	28 *(47)*	21 *(49)*	16 *(41)*	9 (41)	0 *(0)*	0 *(0)*
65–74	24 *(11)*	14 *(8.0)*	3 *(5)*	2 *(5)*	7 *(18)*	3 (14)	0 *(0)*	0 *(0)*
Total	226	165	60	43	39	22	5	1
Sex								
Male	144 *(64)*	101 *(62)*	38 *(63)*	34 *(79)*	26 *(67)*	15 *(65)*	2 *(40)*	0 *(0)*
Female	82 *(36)*	63 *(38)*	22 *(37)*	9 *(21)*	13 *(33)*	8 *(35)*	3 *(60)*	1 *(100)*
Total	226	164	60	43	39	23	5	1
Practice area (overlap n)								
Healthcare	104 *(35)*	81 *(37)*	20 *(27)*	21 *(39)*	9 *(19)*	4 *(13)*	2 *(25)*	0 *(0)*
Industry	108 *(36)*	70 *(32)*	34 *(47)*	21 *(39)*	3 *(6)*	1 *(3)*	4 *(50)*	0 *(0)*
Academic	38 *(13)*	28 *(13)*	8 *(11)*	7 *(13)*	31 *(66)*	21 *(70)*	1 *(12.5)*	0 *(0)*
Other	49 *(16)*	41 *(19)*	11 *(15)*	5 *(9)*	4 *(9)*	4 *(13)*	1 *(12.5)*	1 *(100)*
Total	299	220	73	54	47	30	8	1
Years of experience (years)	Mean ± SD (min-max)	Mean ± SD (min-max)	Mean ± SD (min-max)	Mean ± SD (min-max)	Mean ± SD (min-max)	Mean ± SD (min-max)	Mean ± SD (min-max)	Mean ± SD (min-max)
	18.74 ± 9.98 (1–45)	19.94 ± 10.07 (0–40)	21.29 ± 9.03 (5–50)	22.37 ± 10.44 (4–45)	25.23 ± 9.30 (5–45)	26.22 ± 11.12 (2–44)	6.20 ± 5.50 (2–15)	20 (only 1 trainee)

Across all analysis groups the majority of OPs were male. Similarly, the majority of OPs were in the 45–64 age range for all professional groupings (Table 1). In terms of experience, the Academic/Physician’ group had the most years of experience, followed by Manager/Physician, Physician and last the Trainee group. Area of practise follows the pattern of the groupings, with the majority of the Physician and Manager/Physician groups being between the healthcare and industry sectors, the majority of the ‘Academic/Physician’ being in the academic sector.

Statistical analysis did not identify any statistical significant differences in the distributions of gender (*p* > 0.05), age group (*p* > 0.05), job practice (*p* > 0.05), or years of experience (*p* > 0.05) between round 1 and 2 respondents within each occupational group.

### Round 1-rating

All 12 domains were considered important (90% and above ‘yes’ response) for all the professional groups (see Additional file 1: Table S1). A small number of OPs indicated that some categories were not relevant competencies for their field of practice. ‘Teaching and Educational supervision’ were not considered relevant competencies for 5% of the Physician group. ‘Management skills’ was similarly not considered a relevant competency for 5% the Academic/Physician group either. ‘Research methods’ was considered not relevant for 7% for the Manager/Physician group.

Respondents were asked to rate each subsection of the principle domains on a five-point Likert scale ranging from least important (rating: 1) to absolutely necessary (rating: 5). For each principle domain the subsection scores were averaged and then an overall principle domain average was estimated (Table 2). For all three groups ‘Good Clinical Care’ was the principle domain with the highest score in terms of importance (Table 2). ‘Health Promotion’ scored the lowest for the Physician group and was the lowest along with ‘Teaching and Educational supervision’ for the Manager/Physician group. ‘Teaching and Educational supervision’ was also the lowest scoring group for the Academic/Physician group. Physicians and Manager/Physicians had the best agreement when principle domains are ranked based on the average rating score (Table 2), while Academic/Physicians diverged from the other two groupings.

**Table 2:** Mean Rating (Round 1) Scores of Principle Domains

		Mean Scores				
Rating Score	Principle Domains	All	Physician	Manager/Physician	Academic/Physician	Trainee
1	General Principles of assessment & Management of Occupational Hazards to Health	4.32 ± 0.45	4.32 ± 0.46	4.28 ± 0.47	4.32 ± 0.43	**4.64 ± 0.36**
2	Assessment of disability and fitness for work	4.16 ± 0.23	4.20 ± 0.22	4.14 ± 0.31	4.02 ± 0.27	4.43 ± 0.33
3	Health Promotion	3.75 ± 0.21	3.72 ± 0.17	3.78 ± 0.30	3.93 ± 0.28	3.67 ± 0.29
4	Ethical and Legal issues	4.39 ± 0.28	4.38 ± 0.29	4.41 ± 0.28	4.38 ± 0.26	4.50 ± 0.21
5	Clinical Governance/Clinical Improvement	4.20 ± 0.19	4.20 ± 0.19	4.16 ± 0.27	4.27 ± 0.18	4.00 ± 0.36
6	Communication skills	4.34 ± 0.27	4.35 ± 0.27	4.35 ± 0.30	4.27 ± 0.28	4.46 ± 0.19
7	Team working and leadership skills	4.22 ± 0.13	4.22 ± 0.14	4.25 ± 0.09	4.10 ± 0.14	4.33 ± 0.16
8	Management skills	3.94 ± 0.20	3.89 ± 0.19	4.06 ± 0.26	3.94 ± 0.26	4.21 ± 0.34
9	Environmental Issues Related to Work Practice	3.97 ± 0.12	3.94 ± 0.21	3.99 ± 0.11	3.98 ± 0.19	4.20 ± 0.28
10	Teaching & Educational Supervision	3.80 ± 0.09	3.77 ± 0.08	3.78 ± 0.14	3.88 ± 0.10	4.19 ± 0.37
11	Good Clinical Care	**4.56 ± 0.20**	**4.55 ± 0.20**	**4.63 ± 0.16**	**4.54 ± 0.24**	4.48 ± 0.21
12	Research methods	3.89 ± 0.29	3.87 ± 0.27	3.81 ± 0.37	4.01 ± 0.30	4.25 ± 0.35

Bold items indicate the highest rated principle domain

### Intra-group rating

Comparison of the relative importance to respondents within each group of the 12 principle domains were made by the Wilcoxon signed rank test. The results of these tests are shown in Tables 3, 4, and 5. The results show that for all three groups, Good Clinical Care was always considered most important when compared with the other principle domains. For the Physician and Manager/Physician groups the domain of Health Promotion was always considered least important, while ‘Environmental issues related to work’ and ‘Assessment of disability & fitness for work’ were often not considered as important as the other domains (Tables 3 and 4). For the Academic/Physician the domain almost always considered least important was the ‘Assessment of disability & fitness for work’ (Table 5), while the ‘Environmental issues related to work’ and ‘Health Promotion’ domains were often considered less important than the other domains.

**Table 3:** Results of Wilcoxon signed ranks tests for ‘Physician’ group with mean scores of principle domains indicated

Principle Domains	Good Clinical Care	General Principles of assessment & Management of Occup Health Hazards	Assessment of disability & fitness for work	Environ Issues Related to Work Practice	Health Promotion	Ethical & Legal issues	Clinical Governance & Improvement	Communication skills	Team working & leadership	Teaching & Educational Supervision	Research methods	Management skills
	4.55 ± 0.20	4.32 ± 0.46	4.20 ± 0.22	3.94 ± 0.21	3.72 ± 0.17	4.38 ± 0.29	4.20 ± 0.19	4.35 ± 0.27	4.22 ± 0.14	3.77 ± 0.08	3.87 ± 0.27	3.89 ± 0.19
Good Clinical Care 4.55 ± 0.20		***	***	***	***	***	***	***	***	***	***	***
General Principles of assessment & Management of Occup Health Hazards 4.32 ± 0.46			**	***	***	NS	**	NS	*	***	***	***
Assessment of disability & fitness for work 4.20 ± 0.22				***	***	NS	NS	NS	NS	***	***	***
Environmental Issues Related to Work Practice 3.94 ± 0.21					***	NS	NS	NS	NS	**	NS	NS
Health Promotion 3.72 ± 0.17						NS	NS	NS	NS	NS	NS	NS
Ethical and Legal issues 4.38 ± 0.29							***	NS	***	***	***	***
Clinical Governance & Improvement 4.20 ± 0.19								NS	NS	***	***	***
Communication skills 4.35 ± 0.27									***	***	***	***
Team working &leadership 4.22 ± 0.14										***	***	***
Teaching & Educational Supervision 3.77 ± 0.08											NS	NS
Research methods 3.87 ± 0.27												NS
Management skills 3.89 ± 0.19												

*p < 0.05; **p < 0.01; ***p < 0.001; alternative = “greater”; row item higher versus column

**Table 4:** Results of Wilcoxon signed ranks tests for ‘Manager/Physician’ group with mean scores of principle domains indicated

Principle Domains	Good Clinical Care	General Principles of assessment & Management of Occup Health Hazards	Assessment of disability & fitness for work	Environ Issues Related to Work Practice	Health Promotion	Ethical & Legal issues	Clinical Governance & Improvement	Communication skills	Team working & leadership	Teaching & Educational Supervision	Research methods	Management skills
	4.55 ± 0.20	4.32 ± 0.46	4.20 ± 0.22	3.94 ± 0.21	3.72 ± 0.17	4.38 ± 0.29	4.20 ± 0.19	4.35 ± 0.27	4.22 ± 0.14	3.77 ± 0.08	3.87 ± 0.27	3.89 ± 0.19
Good Clinical Care 4.55 ± 0.20		***	***	***	***	***	***	***	***	***	***	***
General Principles of assessment & Management of Occup Health Hazards 4.32 ± 0.46			*	***	***	NS	NS	NS	NS	***	***	**
Assessment of disability & fitness for work 4.20 ± 0.22				NS	***	NS	NS	NS	NS	***	***	NS
Environ Issues Related to Work Practice 3.94 ± 0.21					*	NS	NS	NS	NS	*	*	NS
Health Promotion 3.72 ± 0.17						NS	NS	NS	NS	NS	NS	NS
Ethical and Legal issues 4.38 ± 0.29							***	NS	*	***	***	***
Clinical Governance & Improvement 4.20 ± 0.19								NS	NS	***	***	*
Communication skills 4.35 ± 0.27									*	***	***	***
Team working & leadership 4.22 ± 0.14										***	***	***
Teaching & Educational Supervision 3.77 ± 0.08											NS	NS
Research methods 3.87 ± 0.27												NS
Management skills 3.89 ± 0.19												

*p < 0.05; **p < 0.01; ***p < 0.001; alternative = “greater”; row item higher versus column

**Table 5:** Results of Wilcoxon signed ranks tests for ‘Academic/Physician’ group with mean scores of principle domains indicated

Principle domains	Good clinical care	General principles of assessment & management of occup health hazards	Assessment of disability & fitness for work	Environ issues related to work practice	Health promotion	Ethical & legal issues	Clinical governance & improvement	Communication skills	Team working & leadership	Teaching & educational supervision	Research methods	Management skills
	4.55 ± 0.20	4.32 ± 0.46	4.20 ± 0.22	3.94 ± 0.21	3.72 ± 0.17	4.38 ± 0.29	4.20 ± 0.19	4.35 ± 0.27	4.22 ± 0.14	3.77 ± 0.08	3.87 ± 0.27	3.89 ± 0.19
Good Clinical Care 4.55 ± 0.20		**	***	***	***	*	***	***	***	***	***	***
General Principles of assessment & Management of Occup Health Hazards 4.32 ± 0.46			**	**	**	NS	NS	NS	**	***	**	***
Assessment of disability & fitness for work 4.20 ± 0.22				NS	NS	NS	NS	NS	NS	*	NS	NS
Environmental Issues Related to Work Practice 3.94 ± 0.21				NS	*	***	**	***	NS	NS	NS	NS
Health Promotion 3.72 ± 0.17					*	***	***	***	NS	NS	NS	NS
Ethical and Legal issues 4.38 ± 0.29						***	NS	**	**	***	***	***
Clinical Governance & Improvement 4.20 ± 0.19							NS	NS	*	***	**	***
Communication skills 4.35 ± 0.27								NS	*	***	***	***
Team working & leadership 4.22 ± 0.14										***	NS	**
Teaching & Educational Supervision 3.77 ± 0.08											NS	NS
Research methods 3.87 ± 0.27												
Management skills 3.89 ± 0.19												

*p < 0.05; **p < 0.01; ***p < 0.001; alternative = “greater”; row item higher versus column

### Round 2-ranking

Two hundred thirty-two responses to the second questionnaire were received (Table 1) [10]. The OPs were 64.9% male and 35.1% female. The majority of respondents (69.7%) were aged 45–64. The distribution by job category was 71.1% Physicians, 18.5% Manager/Physicians, 9.9% Academic/Physicians and 0.4% Trainees. Overall, the mean value of years of experience was 21.0 ± 10.3 years. The Academic/Physician group had the highest years of experience (26.2 ± 11.1 years), followed by the Manager/Physicians (22.4 ± 10.4 years) and then the Physician group (19.9 ± 10.1 years). There were no statistically significant differences in the distributions of gender, age group, job practice, years of experience between the respondents of the first and second rounds [10].

**Fig. 1:**
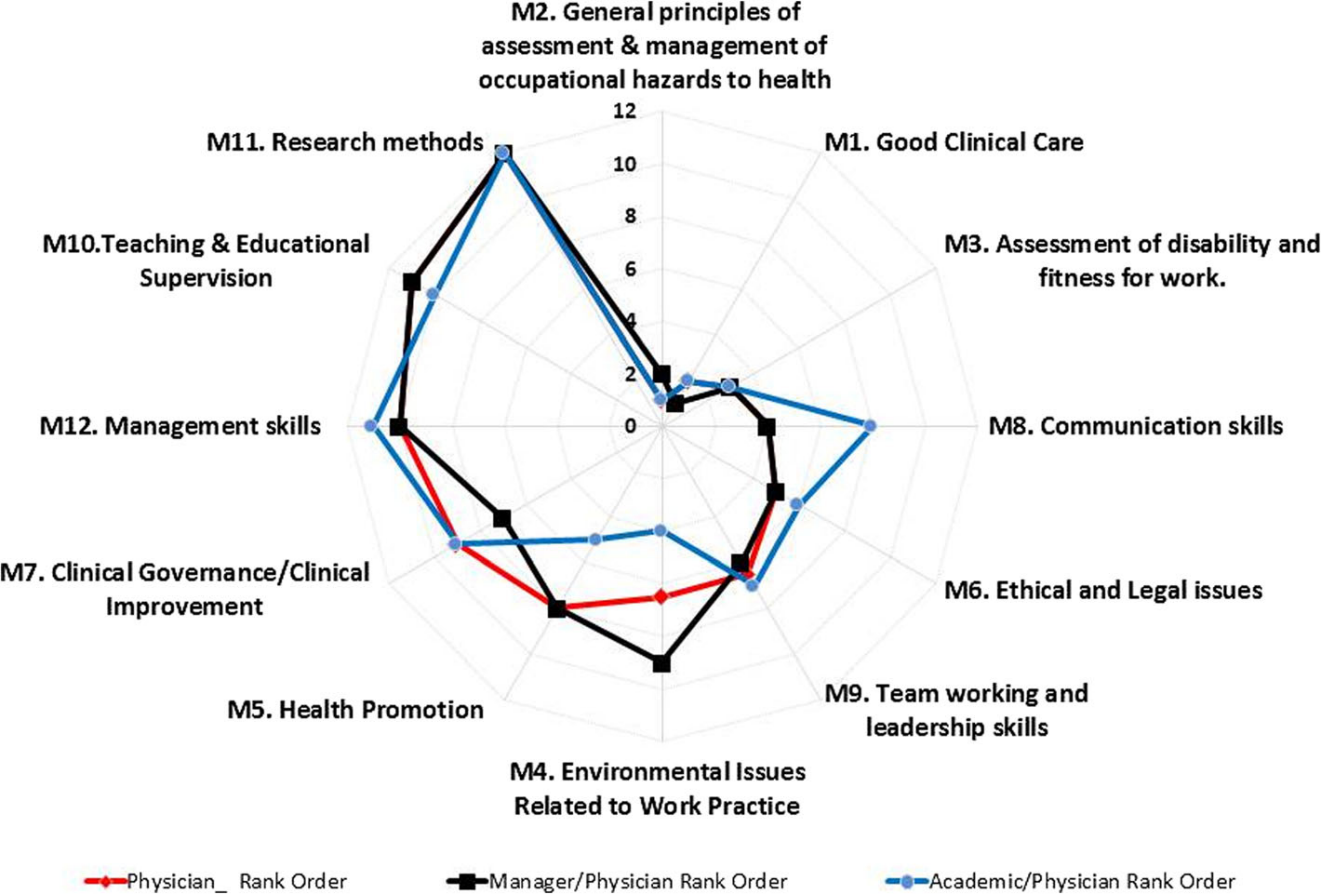
Principle domain ranks (Round 2) for Physicians, Manager/Physicians and Academics/Physicians

The standardised mean rank score was used to obtain the overall rank of the principle domains (Additional file 1: Table S2) and the radar chart in Fig. 1 demonstrates the agreement/disagreement observed in the principle domain ranking for the three groups. Points closest to the centre are of increasing importance. Figure 1 shows that there is good agreement between the Physician and Manager/Physician groups, and the group deviating the most is the Academic/Physicians. The top three and bottom three principle domains are in good agreement across all groups. The principle domains with the most variance were *‘Communication Skills’* (ranked fourth for the Physician and the Manager/Physician groups but eighth for the Academic/Physician group); ‘*Environmental Issues related to Work Practice’* (ranked in 6-7th place for Physicians, 9th for Manager/Physicians and 4th for Academic/Physicians); ‘*Health Promotion*’ (ranked 8th for the Physician and Manager/Physician groups but 5th for the Academic/Physician group); and ‘*Clinical Governance/Clinical Improvement’* (ranked 9th for the Physicians and the Academic/Physicians but 7th for the Manager/Physicians).

The principle domain subcategory ranking reflects what each group considered as the highest principle domain. For Physicians and Academic/Physicians the subcategory ‘B1. Understand and apply the principles of risk assessment- i.e. recognition of potential hazards in the work environment, evaluating risks and providing advice and information on control measures’ was ranked the highest subcategory (Additional file 1: Table S3). Furthermore, these two occupational groups had four subcategories from the ‘General principles of assessment & management of occupational hazards to health’ (i.e. B1, B3, B7 and B2) domain featured in their top five subcategories. For the Manager/Physician group the subcategory ‘A2. Take and analyse a clinical and occupational history including an exposure history in a relevant, succinct and systematic manner’ was ranked as the most important category. The top five subcategories for the Manager/Physician group were from the ‘Good Clinical Care’ domain (A2, A1, A4, A3), apart from one ‘C1. Assessing and advising on impairment, disability and fitness for work’ (Additional file 1: Table S2). The top ten subcategories demonstrate considerable variability. Four out of the ten (B1, A2, B2, C1), all feature within the top ten subcategories in various rankings within the three groups, and six subcategories (B5, A4, A3, A8, A5, and A6) only feature in the top ten of one group (Additional file 1: Table S3).

## Discussion

### Summary of findings

In this study, we compared OPs views on competencies by field of practice. By consensus, all the competency domains were regarded as important by respondents with ‘yes’ scores of 90% and over in all 12 identified domains. In general, there was good agreement between the Physician and Manager/Physician groups and the group deviating the most was the Academic/Physicians. The top three and bottom three principle domains were in good agreement across all groups. All groups were in agreement on the top 3 ranked principle domains. These were all clinically based and what would be considered core OH activities, namely ‘General principles of assessment & management of occupational hazards to health’, ‘Good Clinical Care’ and ‘Assessment of disability and fitness for work’. There was similar agreement in the bottom 3 ranked principle domains, which included management and academic related competencies (‘Management skills’, ‘Teaching & Educational Supervision’, ‘Research methods’). The principle domains with considerable intergroup variance were ‘Environmental Issues Related to Work Practice’ and ‘Communication Skills’. The variance in the first of these domains may reflect the direct importance of environmental issues for understanding and assessing exposures and impacts for research (Academic/Physicians) and practice (Physicians) purposes. OPs with managerial roles are very likely to be further removed from direct pathways of exposure and impact, and this is translated by the lower rank. The latter can be explained by the fact that the Physicians and Manager/Physicians are in more ‘front facing’ roles regularly engaging with a range of stakeholders (including employees, employers, administrative staff and other clinicians within the multi-disciplinary OH team), for which effective communication will be essential/inherent. Furthermore, being fundamentally clinical, and with communication and the clinician-patient relationship increasingly recognised as important determinants of patient satisfaction [21] and improved clinical outcomes [22, 23], these two groups require to demonstrate advanced competence and skills in this domain and to ensure continuing development of this skill throughout their careers [24–27].

The analysis of the subsection priorities demonstrated that for Physicians and Academic/Physicians ‘Understanding and applying the principles of risk assessment was ranked the highest subcategory, whereas the Manager/Physician group had a more operational, and service delivery orientated focus with ‘Taking and analysing a clinical and occupational history including an exposure history in a relevant, succinct and systematic manner’ ranked as the most important category.

No obvious bias was observed amongst managers and academics towards the competency set most applicable to their practice, with ‘*Management skills’* ranked 10th by Manager/Physician groups and ‘*Research methods’* ranked 12th by the Academic/Physician group.

The principle domain of ‘*Research Methods’* was the least important category across all three OP professional groups (Table 2) and was considered not relevant for 7% of the Manager/Physician group. A recent study by Heikkila et al. (2015) of Finnish doctors demonstrated that the ‘opportunity to carry out research’ was one of the least favourable motives for choosing a specialty and this was less important for female doctors [2]. Hoving et al. [28] showed that “physicians are inclined to use evidence based medicine and believe that the use of evidence based medicine improved the quality and attractiveness of their work” and Salter and Kothari [29] state that practice based on sound research is often considered best practice, and allows for decision making to be part of a “logical, explicit, transparent, and measurable process”. Our findings reflect that while respondents consider direct involvement in research to be of low priority, they acknowledge the importance of an evidence base in their clinical practice. This supports the need to maintain a strong academic base for the speciality.

### Strengths and limitations

#### Strengths

Previous studies have assessed the professional development of OPs in terms of practice competencies and curricula, but to our knowledge this is the first study to examine inter-OP group differences by job/field of practice. Furthermore, consensus was been derived from OPs working across a range of countries both developed and developing and with a large spectrum of expertise, ranging from newly specialised OPs to highly experienced (years of expertise ranging from 0 to 50 years of experience). Furthermore, we previously demonstrated that rank comparisons between continents were highly correlated as well by age, gender and years of experience [10].

#### Limitations

The relatively low response rate is a limitation of our study and earlier comparable studies have reported similar challenges [30–32]. Language barriers may have been an influencing factor. The stronger European response [10] may also bias the wider representativeness of our findings but the questionnaire was distributed across a range of networks and the sources of response were beyond our control. The low number of trainee responses did not allow any meaningful conclusions to be drawn and could feasibly be explained by the distribution networks, with members more likely being established OPs. A comparison of trainees views on competency requirements with those experienced in different areas of occupational health practice could provide invaluable information on potential knowledge gaps in current curricula. It is important to acknowledge that there was a high degree of crossover in OH practice and the categorisation process was based on self-reported job titles and not on specific job roles and tasks. Although management functions may be the primary role of a Manager they may also be involved in some teaching or research activities. Likewise, Academic/Physicians may predominantly be undertaking research and teaching work but they may have some management and clinical roles as well.

### Comparison with previous studies

Given its novel perspective, there are no published studies for direct comparison with our study. Our findings however are consistent with those of other studies notably the earlier Macdonald et al. [6, 13]. European study where ‘occupational hazards to health’ was the highest ranked principle domain. In Macdonald et al. [6] however, ‘research methods’, were considered a higher priority, ranked as fourth. ‘Law & ethics’, although ranked out with the top 3 in our study, ranked second highest in Macdonald et al. [6] and highest in a study of UK customers views on required OH competencies [15]. The intra-group comparison results of our study, showed that ‘environmental issues related to work practice’ were often least important within respondents of a group and ‘health promotion’ was least important for the Physician and Manager/Physician group but varied for the Academic/Physician group. This is in line with the findings of the European study [6], which showed that environmental medicine was significantly less important and health promotion showed greater variation across the subsections. It is difficult to compare the findings of Delclos et al. [4] study due to design and classification differences. In that study, the competency skill sets reported most commonly were administrative/management (health & safety, legal, regulatory considerations) then professional practice (ethical considerations) followed by research [4].

## Conclusion

This study has compared perspectives on the competencies required of OPs, by field of practice. It has observed that there is general agreement between the three OP professional groups. The group diverging the most in opinion is the Academic/Physicians, while good general agreement is depicted between the Physician and Manager/Physician groups. Recognition of these perspective differences by OP professional group is important for those directly involved in defining and developing competency standards, particularly if these tasks continue to a large extent to be undertaken by academics [8]. The findings of this study can help to fine tune, following basic OP training, more focussed and tailored training programmes with emphasis on the bespoke skills and competencies (for example, more advanced communication training for the Physicians and Manager/Physicians and more comprehensive Environmental impact training for the Academic/Physician and Physician groups) required for these specialised fields that are reflective of practice. The international perspective facilitates scope for common training/curricula development at national, regional and international level. Further research opportunities could include qualitative inter-group evaluations exploring the reasons for their higher and lower priority choices.

## Additional file

Additional file 1: Supplementary Material. (DOCX 46 kb)

## Abbreviations

NHS: National Health Service
OH: Occupational health
OM: Occupational medicine
OP: Occupational physician
PD: Principle domain
