# Supplementary material for: Differences in opinions of occupational physicians on the required competencies by field of practice: results of an international Delphi study
Source: BMC Med Educ. 2018 Apr 2;18:62. doi: 10.1186/s12909-018-1139-9 (PMC5879917; doi:10.1186/s12909-018-1139-9)
Supplement: Supplementary file 1 — Supplementary Material. (DOCX 46 kb) [file 12909_2018_1139_MOESM1_ESM.docx]

Differences in opinions of occupational physicians on the required competencies by field of practice: results of an international Delphi study

**[Supplementary Material]**

Evangelia Demou^1,*^, Drushca Lalloo^2^, Ewan B. Macdonald^2^

1. MRC/CSO Social and Public Health Sciences Unit, Institute of Health and Wellbeing, University of Glasgow, Glasgow, G2 3QB
2. Healthy Working Lives Group, Institute of Health and Wellbeing, University of Glasgow, Glasgow, G12 8RZ

*Corresponding Author

Dr Evangelia Demou

MRC/CSO Social and Public Health Sciences Unit,

Institute of Health and Wellbeing,

University of Glasgow,

Glasgow, G2 3QB

Email: [Evangelia.demou@glasgow.ac.uk](mailto:Evangelia.demou@glasgow.ac.uk)

Tel: 0141 353 7580

**Table S1.** Priorities in Principle Domains. Rating Round Results

|  |  | **Physician** | | | **Manager/Physician** | | | **Academic/Physician** | | | **Trainee** | | |
| --- | --- | --- | --- | --- | --- | --- | --- | --- | --- | --- | --- | --- | --- |
| **Rating Score** | **Principle Domains** | **Yes (%)** | **No (%)** | **Not relevant (%)** | **Yes (%)** | **No (%)** | **Not relevant (%)** | **Yes (%)** | **No (%)** | **Not Relevant (%)** | **Yes (%)** | **No (%)** | **Not relevant (%)** |
| **1** | General Principles of assessment & Management of Occupational Hazards to Health | **226 *(99.1)*** | 2  *(0.9)* | 0  *(0)* | **60**  ***(100)*** | 0  *(0)* | 0  *(0)* | 38  *(97.4)* | 1  *(2.6)* | 0  *(0)* | **5**  ***(100)*** | 0  *(0)* | 0  (0) |
| **2** | Assessment of disability and fitness for work | 220 *(96.5)* | 3  *(1.3)* | 5  *(2.2)* | **60**  ***(100)*** | 0  *(0)* | 0  *(0)* | 37  *(94.9)* | 2  *(5.1)* | 0  *(0)* | 4  *(80)* | 1  *(20)* | 0  *(0)* |
| **3** | Health Promotion | 213 *(93.4)* | 9  *(3.9)* | 6  *(2.6)* | **60**  ***(100)*** | 0  *(0)* | 0  *(0)* | 38  *(97.4)* | 1  *(2.6)* | 0  *(0)* | 4  *(80)* | 1  *(20)* | 0  *(0)* |
| **4** | Ethical and Legal issues | 223 *(97.8)* | 1  *(0.4)* | 4  *(1.8)* | 59  *(98.3)* | 1  *(1.7)* | 0  *(0)* | **39**  ***(100)*** | 0  *(0)* | 0  *(0)* | **5**  ***(100)*** | 0  *(0)* | 0  *(0)* |
| **5** | Clinical Governance/Clinical Improvement | 212 *(93.0)* | 7  *(3.1)* | 9  *(3.9)* | 58  *(96.7)* | 1  *(1.7)* | 1  *(1.7)* | 38  *(97.4)* | 0  *(0)* | 1  *(2.6)* | **5**  ***(100****)* | 0  *(0)* | 0  *(0)* |
| **6** | Communication skills | 223 *(97.8)* | 2  *(0.9)* | 3  *(1.3)* | **60**  ***(100)*** | 0  *(0)* | 0  *(0)* | **39**  ***(100)*** | 0  *(0)* | 0  *(0)* | **5**  ***(100)*** | 0  *(0)* | 0  *(0)* |
| **7** | Team working and leadership skills | 219 *(96.1)* | 2  *(0.9)* | 7  *(3.1)* | **60**  ***(100)*** | 0  *(0)* | 0  *(0)* | 38  *(97.4)* | 1  *(2.6)* | 0  *(0)* | **5**  ***(100)*** | 0  *(0)* | 0  *(0)* |
| **8** | Management skills | 209 *(91.7)* | 7  *(3.1)* | 12  *(5.3)* | 57  *(95.0)* | 2  *(3.3)* | 1  *(1.7)* | 36  *(92.3)* | 1  *(2.6)* | 2  *(5.1)* | **5**  ***(100)*** | 0  *(0)* | 0  *(0)* |
| **9** | Environmental Issues Related to Work Practice | 211 *(92.5)* | 9  *(3.9)* | 8  *(3.5)* | 57  *(95.0)* | 3  *(5.0)* | 0  *(0)* | 36  *(92.3)* | 3  *(7.7)* | 0  *(0)* | **5**  ***(100)*** | 0  *(0)* | 0  *(0)* |
| **10** | Teaching & Educational Supervision | 207 *(90.8)* | 9  *(3.9)* | 12  (5.3) | **55**  ***(91.7)*** | 3  *(5.0)* | 2  *(3.3)* | **35**  ***(89.7)*** | 4  *(10.3)* | 0  *(0)* | 4  *(80)* | 1  *(20)* | 0  *(0)* |
| **11** | Good Clinical Care | 214 *(93.9)* | 3  *(1.3)* | 11  *(4.8)* | 58  *(96.7)* | 2  *(3.3)* | 0  *(0)* | 38  *(97.4)* | 1  *(2.6)* | 0  *(0)* | **5**  ***(100)*** | 0  *(0)* | 0  *(0)* |
| **12** | Research methods | **205 *(89.9)*** | 13  *(5.7)* | 10  *(4.4)* | **55**  ***(91.7)*** | 1  *(1.7)* | 4  *(6.7)* | 37  *(94.9)* | 2  *(5.1)* | 0  *(0)* | **3**  ***(60)*** | 2  *(40)* | 0  *(0)* |

**Table S2.** Principle Domain Ranks (Round 2) by self-reported job title (Compare Tables 4& 8 in discussion). Highlighted sections with 2 or more rank order differences

| **Principle Domains** | **Physician Rank Order** | **Manager/**  **Physician**  **Rank Order** | **Academic/**  **Physician**  **Rank Order** |
| --- | --- | --- | --- |
| **M2.** General principles of assessment & management of occupational hazards to health | **1** | 2 | **1** |
| **M1.** Good Clinical Care | 2 | **1** | 2 |
| **M3.** Assessment of disability and fitness for work. | 3 | 3 | 3 |
| **M8.** Communication skills | 4 | 4 | 8 |
| **M6.** Ethical and Legal issues | 5 | 5 | 6 |
| **M9.** Team working and leadership skills | 6&7 | 6 | 7 |
| **M4.** Environmental Issues Related to Work Practice | 6&7 | 9 | 4 |
| **M5.** Health Promotion | 8 | 8 | 5 |
| **M7.** Clinical Governance/Clinical Improvement | 9 | 7 | 9 |
| **M12.** Management skills | 10 | 10 | 11 |
| **M10.**Teaching & Educational Supervision | 11 | 11 | 10 |
| **M11.** Research methods | 12 | 12 | 12 |

**Table S3.** All subdomains ranked by weighed standardised rank score (smallest value=most important; highest value=least important) –[Highlighted in grey top 10 and bottom 10 subcategories]

| **Physician_All subdomains** | **w_Std rank** | **Manager/Physician_All subdomains** | **w_Std Rank** | **Academic/Physician_All subdomains** | **w_Std Rank** |
| --- | --- | --- | --- | --- | --- |
| B1. Understand and apply the principles of risk assessment- ie recognition of potential hazards in the work environment, evaluating risks and providing advice and information on control measures . | 2.1 | A2. Take and analyse a clinical and occupational history including an exposure history in a relevant, succint and systematic manner | 2.1 | B1. Understand and apply the principles of risk assessment- ie recognition of potential hazards in the work environment, evaluating risks and providing advice and information on control measures . | 2.7 |
| B2. Provide advice on medical aspects/ factors relevant to the risk assessment. | 3.3 | A1. Broad general medical knowledge of all common medical conditions including diagnostic criteria, evidence based treatment spectrum and prognosis | 3.2 | B3. Carry out a workplace visit and produce a report. | 3.7 |
| B7. Identify work related ill health and provide advice on prognosis, prevention and management. | 3.6 | C1. Assessing and advising on impairment, disability and fitness for work | 4.3 | B7. Identify work related ill health and provide advice on prognosis, prevention and management. | 3.9 |
| B3. Carry out a workplace visit and produce a report. | 4.2 | A4. Interpret the results of investigations, including especially those relating to occupational attribution and functional prognosis | 4.6 | A2. Take and analyse a clinical and occupational history including an exposure history in a relevant, succint and systematic manner | 4.1 |
| B4. Understand the core principles of occupational/ industrial hygiene and be able to interpret reports and related measurements. | 4.6 | A3. Perform a reliable and appropriate examination | 4.6 | B2. Provide advice on medical aspects/ factors relevant to the risk assessment. | 4.3 |
| C1. Assessing and advising on impairment, disability and fitness for work | 4.6 | B1. Understand and apply the principles of risk assessment- ie recognition of potential hazards in the work environment, evaluating risks and providing advice and information on control measures . | 4.8 | B8. Carry out and evaluate health surveillance including biological monitoring for workers exposed to occupational hazards. | 4.5 |
| A2. Take and analyse a clinical and occupational history including an exposure history in a relevant, succint and systematic manner | 4.8 | B2. Provide advice on medical aspects/ factors relevant to the risk assessment. | 5.6 | B4. Understand the core principles of occupational/ industrial hygiene and be able to interpret reports and related measurements. | 4.8 |
| B5. Understand the core principles of ergonomics and be able to interpret reports. | 5.4 | A8. Appreciate the importance and interaction of psychological and social factors in patient’s disease and illness behaviour. | 6.4 | C1. Assessing and advising on impairment, disability and fitness for work | 5.1 |
| B8. Carry out and evaluate health surveillance including biological monitoring for workers exposed to occupational hazards. | 5.8 | A5. Perform investigations competently where relevant | 6.7 | B6. Understand the core principles of toxicology and be able to interpret reports and related measurements. | 5.5 |
| B6. Understand the core principles of toxicology and be able to interpret reports and related measurements. | 5.9 | A6. Record concisely, accurately, confidentially and legibly all medical records, and date and sign all records | 6.7 | A1. Broad general medical knowledge of all common medical conditions including diagnostic criteria, evidence based treatment spectrum and prognosis | 6.4 |
| H1. Be able to communicate effectively both orally and in writing to patients and other stakeholders in a manner that they understand | 7.2 | A7. Show empathy with and listen to employee. | 6.8 | B5. Understand the core principles of ergonomics and be able to interpret reports. | 6.4 |
| A1. Broad general medical knowledge of all common medical conditions including diagnostic criteria, evidence based treatment spectrum and prognosis | 7.3 | H1. Be able to communicate effectively both orally and in writing to patients and other stakeholders in a manner that they understand | 6.9 | B9. Evaluate and advise on first aid facilities in the workplace. | 7.3 |
| B9. Evaluate and advise on first aid facilities in the workplace. | 8.1 | B7. Identify work related ill health and provide advice on prognosis, prevention and management. | 7.7 | B10. Advising on disaster and contingency planning within the organisation. | 8.5 |
| B10. Advising on disaster and contingency planning within the organisation. | 8.6 | A9. Manage time and problems effectively. | 8.4 | B11. Understand the principles of Travel Medicine | 8.8 |
| B11. Understand the principles of Travel Medicine | 9.1 | B4. Understand the core principles of occupational/ industrial hygiene and be able to interpret reports and related measurements. | 8.9 | D1. Recognise and advise on health risks in the general environment arising from industrial activities. | 9.0 |
| A3. Perform a reliable and appropriate examination | 9.3 | B3. Carry out a workplace visit and produce a report. | 9.9 | A3. Perform a reliable and appropriate examination | 9.5 |
| A7. Show empathy with and listen to employee. | 10.5 | B8. Carry out and evaluate health surveillance including biological monitoring for workers exposed to occupational hazards. | 10.0 | A4. Interpret the results of investigations, including especially those relating to occupational attribution and functional prognosis | 11.2 |
| A4. Interpret the results of investigations, including especially those relating to occupational attribution and functional prognosis | 10.7 | B5. Understand the core principles of ergonomics and be able to interpret reports. | 11.4 | A5. Perform investigations competently where relevant | 11.6 |
| A8. Appreciate the importance and interaction of psychological and social factors in patient’s disease and illness behaviour. | 11.8 | B6. Understand the core principles of toxicology and be able to interpret reports and related measurements. | 11.5 | A6. Record concisely, accurately, confidentially and legibly all medical records, and date and sign all records | 12.1 |
| C2. Advising on rehabilitation | 12.1 | C5. Assessing and advising on sickness absence | 12.1 | A7. Show empathy with and listen to employee. | 12.1 |
| A6. Record concisely, accurately, confidentially and legibly all medical records, and date and sign all records | 13.3 | F1. Be well-informed about acts, regulations, codes of practice and guidance relevant to the workplace setting. | 12.3 | C2. Advising on rehabilitation | 12.3 |
| C5. Assessing and advising on sickness absence | 14.3 | C2. Advising on rehabilitation | 13.3 | C3. Advising on redeployment | 12.4 |
| A5. Perform investigations competently where relevant | 14.6 | C3. Advising on redeployment | 14.4 | C5. Assessing and advising on sickness absence | 13.6 |
| F1. Be well-informed about acts, regulations, codes of practice and guidance relevant to the workplace setting. | 14.7 | H3. Demonstrate effective consultation skills. | 16.3 | E1. Assessing needs for health promotion in a workforce. | 13.8 |
| C3. Advising on redeployment | 14.7 | B10. Advising on disaster and contingency planning within the organisation. | 16.6 | H1. Be able to communicate effectively both orally and in writing to patients and other stakeholders in a manner that they understand | 13.9 |
| H3. Demonstrate effective consultation skills. | 16.5 | B9. Evaluate and advise on first aid facilities in the workplace. | 17.0 | A8. Appreciate the importance and interaction of psychological and social factors in patient’s disease and illness behaviour. | 14.7 |
| A9. Manage time and problems effectively. | 16.7 | H2. Prepare written reports on a range of topics for a range of groups including managers, unions (e.g. for safety representatives) and health professionals. | 17.1 | F1. Be well-informed about acts, regulations, codes of practice and guidance relevant to the workplace setting. | 15.4 |
| F5. Respect the patient’s right to confidentiality. | 17.4 | C10. Liaising with other health professionals in assessing capability for work | 17.1 | C10. Liaising with other health professionals in assessing capability for work | 15.8 |
| C10. Liaising with other health professionals in assessing capability for work | 17.7 | B11. Understand the principles of Travel Medicine | 17.5 | A9. Manage time and problems effectively. | 17.4 |
| H2. Prepare written reports on a range of topics for a range of groups including managers, unions (e.g. for safety representatives) and health professionals. | 18.0 | G1. Practise evidence based medicine. | 17.5 | I1. Understand how a team works effectively | 17.4 |
| C4. Assessing and advising on early retirement due to ill-health | 18.6 | F5. Respect the patient’s right to confidentiality. | 17.6 | D2. Assess and advise on the control of environmental exposures from the workplace. | 18.1 |
| H8. Apply ethical principles when communicating with others about individuals. | 19.3 | C8. Assessing the ageing worker and advising on work ability | 18.2 | C4. Assessing and advising on early retirement due to ill-health | 18.9 |
| C8. Assessing the ageing worker and advising on work ability | 20.2 | H8. Apply ethical principles when communicating with others about individuals. | 18.7 | C8. Assessing the ageing worker and advising on work ability | 20.0 |
| C6. Advising on legal issues related to disability | 20.3 | C7. Advising on drug and alcohol problems | 20.0 | C6. Advising on legal issues related to disability | 21.8 |
| C7. Advising on drug and alcohol problems | 20.4 | C4. Assessing and advising on early retirement due to ill-health | 20.4 | C7. Advising on drug and alcohol problems | 21.8 |
| D1. Recognise and advise on health risks in the general environment arising from industrial activities. | 20.5 | C6. Advising on legal issues related to disability | 20.4 | I2. Be an effective team player | 22.9 |
| I1. Understand how a team works effectively | 20.5 | I1. Understand how a team works effectively | 21.3 | D6. Identify and manage concerns about the health effects of human exposure to environmental hazards eg. toxins and pollutants. | 23.4 |
| G1. Practise evidence based medicine. | 21.4 | H5. Anticipate potential problems. | 22.0 | C9. Advising on vocational rehabilitation | 23.5 |
| H5. Anticipate potential problems. | 22.0 | I2. Be an effective team player | 23.3 | F5. Respect the patient’s right to confidentiality. | 24.8 |
| C9. Advising on vocational rehabilitation | 22.2 | E1. Assessing needs for health promotion in a workforce. | 24.7 | D3. Understand when to obtain environmental monitoring. | 25.3 |
| E1. Assessing needs for health promotion in a workforce. | 23.7 | C9. Advising on vocational rehabilitation | 24.8 | J1. Identify learning outcomes and construct educational objectives. | 26.6 |
| I2. Be an effective team player | 24.8 | L2. Be able to strategically plan and set objectives for delivering an occupational health service. | 25.8 | D5. Effectively communicate risk from various environmental exposures including water pollution, hazardous waste, sewage, household chemicals and radiation. | 27.5 |
| H4. Manage dissatisfied patients/ relatives. | 25.2 | D1. Recognise and advise on health risks in the general environment arising from industrial activities. | 26.3 | L1. Be able to understand the principles and practice of management. | 28.2 |
| K1. Be able to define a problem in terms of needs for an evidence base. | 26.8 | H7. Effective presentation skills. Make clear oral presentations to a range of audiences using audiovisual equipment. | 26.5 | D4. Interpret and explain the results of environmental monitoring. | 28.7 |
| D2. Assess and advise on the control of environmental exposures from the workplace. | 26.8 | H4. Manage dissatisfied patients/ relatives. | 26.8 | G1. Practise evidence based medicine. | 29.4 |
| F2. Providing advice to managers, safety representatives and employees of their legal obligations. | 26.9 | F2. Providing advice to managers, safety representatives and employees of their legal obligations. | 27.4 | K2. Be able to undertake a literature search. | 29.4 |
| L2. Be able to strategically plan and set objectives for delivering an occupational health service. | 27.8 | I4. Demonstrate leadership | 27.6 | L2. Be able to strategically plan and set objectives for delivering an occupational health service. | 29.4 |
| H7. Effective presentation skills. Make clear oral presentations to a range of audiences using audiovisual equipment. | 28.3 | H6. Be able to effectively participate in committees and to act as a chairperson. | 29.6 | E3. Organising, providing and evaluating work related health promotion activities. | 30.4 |
| H6. Be able to effectively participate in committees and to act as a chairperson. | 28.6 | G3. Develop and institute clinical guidelines and integrated care pathways. Be aware of advantages and disadvantages of guidelines. | 31.0 | F2. Providing advice to managers, safety representatives and employees of their legal obligations. | 30.9 |
| J1. Identify learning outcomes and construct educational objectives. | 30.0 | K1. Be able to define a problem in terms of needs for an evidence base. | 32.5 | H2. Prepare written reports on a range of topics for a range of groups including managers, unions (e.g. for safety representatives) and health professionals. | 31.9 |
| F4. Responsibilities relating to data protection. | 32.4 | F4. Responsibilities relating to data protection. | 33.5 | J2. Design and deliver an effective teaching event or short course. | 32.5 |
| H9. Motivational interviewing skills | 33.0 | J1. Identify learning outcomes and construct educational objectives. | 33.7 | K1. Be able to define a problem in terms of needs for an evidence base. | 35.5 |
| L1. Be able to understand the principles and practice of management. | 33.8 | K2. Be able to undertake a literature search. | 33.9 | F4. Responsibilities relating to data protection. | 37.0 |
| D3. Understand when to obtain environmental monitoring. | 34.7 | H9. Motivational interviewing skills | 34.0 | E2. Give advice on nutritional and other healthy lifestyle issues. | 38.3 |
| F3. Evaluate compliance with new legislation | 35.7 | L1. Be able to understand the principles and practice of management. | 35.1 | I4. Demonstrate leadership | 38.8 |
| I3. Understand different leadership styles | 36.1 | F3. Evaluate compliance with new legislation | 36.0 | G2. Be able to handle and deal with complaints in a focused and constructive manner. | 39.3 |
| I4. Demonstrate leadership | 36.6 | I3. Understand different leadership styles | 38.1 | H8. Apply ethical principles when communicating with others about individuals. | 39.3 |
| D6. Identify and manage concerns about the health effects of human exposure to environmental hazards eg. toxins and pollutants. | 37.6 | F6. Understand the process for gaining informed consent for clinical and research activities | 38.3 | H3. Demonstrate effective consultation skills. | 40.9 |
| F6. Understand the process for gaining informed consent for clinical and research activities | 37.9 | E3. Organising, providing and evaluating work related health promotion activities. | 39.0 | G3. Develop and institute clinical guidelines and integrated care pathways. Be aware of advantages and disadvantages of guidelines. | 42.1 |
| K2. Be able to undertake a literature search. | 43.1 | L8. Be able to manage a team. | 39.0 | H5. Anticipate potential problems. | 42.4 |
| I6. Influencing and negotiation | 46.2 | G2. Be able to handle and deal with complaints in a focused and constructive manner. | 39.5 | F3. Evaluate compliance with new legislation | 43.1 |
| E3. Organising, providing and evaluating work related health promotion activities. | 46.6 | J2. Design and deliver an effective teaching event or short course. | 40.4 | K3. Be able to undertake a systematic and critical appraisal of scientific literature. | 43.5 |
| G5. Be able to balance risk and benefits with patients. | 46.7 | I6. Influencing and negotiation | 40.4 | J3.Teach large and small groups effectively. | 44.7 |
| D4. Interpret and explain the results of environmental monitoring. | 46.8 | G5. Be able to balance risk and benefits with patients. | 40.7 | I3. Understand different leadership styles | 44.8 |
| J2. Design and deliver an effective teaching event or short course. | 47.3 | D2. Assess and advise on the control of environmental exposures from the workplace. | 41.4 | F6. Understand the process for gaining informed consent for clinical and research activities | 46.9 |
| K3. Be able to undertake a systematic and critical appraisal of scientific literature. | 47.3 | K3. Be able to undertake a systematic and critical appraisal of scientific literature. | 42.5 | H4. Manage dissatisfied patients/ relatives. | 49.1 |
| D5. Effectively communicate risk from various environmental exposures including water pollution, hazardous waste, sewage, household chemicals and radiation. | 48.3 | G4. Report and investigate critical incidents. | 44.5 | J4. Select and use appropriate teaching resources. | 49.1 |
| L4. Evaluate the effectiveness and quality of an occupational health service. | 48.6 | D6. Identify and manage concerns about the health effects of human exposure to environmental hazards eg. toxins and pollutants. | 45.9 | J5. Give constructive effective feedback. | 49.1 |
| G3. Develop and institute clinical guidelines and integrated care pathways. Be aware of advantages and disadvantages of guidelines. | 49.0 | I5. Delegation | 47.2 | I5. Delegation | 49.7 |
| G2. Be able to handle and deal with complaints in a focused and constructive manner. | 49.6 | L4. Evaluate the effectiveness and quality of an occupational health service. | 48.3 | G5. Be able to balance risk and benefits with patients. | 52.0 |
| L8. Be able to manage a team. | 49.9 | J3.Teach large and small groups effectively. | 51.3 | G4. Report and investigate critical incidents. | 54.8 |
| I5. Delegation | 50.3 | D3. Understand when to obtain environmental monitoring. | 53.1 | H6. Be able to effectively participate in committees and to act as a chairperson. | 55.3 |
| J4. Select and use appropriate teaching resources. | 51.1 | J5. Give constructive effective feedback. | 56.2 | I6. Influencing and negotiation | 57.4 |
| J3.Teach large and small groups effectively. | 53.0 | G6. Be actively involved in clinical audit. | 57.7 | H7. Effective presentation skills. Make clear oral presentations to a range of audiences using audiovisual equipment. | 57.7 |
| J5. Give constructive effective feedback. | 54.9 | J4. Select and use appropriate teaching resources. | 57.9 | L8. Be able to manage a team. | 57.7 |
| L6. Define the roles and responsibilities of staff in providing an occupational health service | 56.0 | L6. Define the roles and responsibilities of staff in providing an occupational health service | 58.4 | L4. Evaluate the effectiveness and quality of an occupational health service. | 58.3 |
| G4. Report and investigate critical incidents. | 56.6 | D5. Effectively communicate risk from various environmental exposures including water pollution, hazardous waste, sewage, household chemicals and radiation. | 64.8 | L6. Define the roles and responsibilities of staff in providing an occupational health service | 59.6 |
| E2. Give advice on nutritional and other healthy lifestyle issues. | 61.7 | D4. Interpret and explain the results of environmental monitoring. | 65.5 | K4. Be able to develop and execute an appropriate study design | 65.0 |
| K4. Be able to develop and execute an appropriate study design | 63.5 | L3. Finance management. Be able to negotiate and manage a budget/resources. | 66.2 | H9. Motivational interviewing skills | 65.5 |
| L3. Finance management. Be able to negotiate and manage a budget/resources. | 64.5 | J9. Deliver effective mentorship | 68.3 | J6. Evaluate programmes and events. | 70.2 |
| J7. Use appropriate assessment methods. | 71.1 | E2. Give advice on nutritional and other healthy lifestyle issues. | 68.3 | L3. Finance management. Be able to negotiate and manage a budget/resources. | 71.3 |
| G6. Be actively involved in clinical audit. | 73.7 | K4. Be able to develop and execute an appropriate study design | 68.3 | J7. Use appropriate assessment methods. | 72.1 |
| L5. Be able to market an occupational health service. | 73.7 | J6. Evaluate programmes and events. | 75.8 | J8. Conduct effective appraisals. | 75.1 |
| J9. Deliver effective mentorship | 75.8 | L5. Be able to market an occupational health service. | 76.4 | J9. Deliver effective mentorship | 75.5 |
| K5. Be able to use databases. | 76.8 | J7. Use appropriate assessment methods. | 76.9 | K5. Be able to use databases. | 78.4 |
| K8. To understand the principles of research ethics and ethical considerations in research. | 77.8 | K8. To understand the principles of research ethics and ethical considerations in research. | 80.2 | G6. Be actively involved in clinical audit. | 79.4 |
| J6. Evaluate programmes and events. | 79.1 | K5. Be able to use databases. | 83.4 | K8. To understand the principles of research ethics and ethical considerations in research. | 81.8 |
| J8. Conduct effective appraisals. | 82.3 | J8. Conduct effective appraisals. | 84.1 | L5. Be able to market an occupational health service. | 83.6 |
| L7. Support formulation of job descriptions. | 85.6 | K6. Be able to carry out basic statistical analyses. | 89.9 | K7. Present investigation and results in the format of a research based report. | 95.9 |
| K6. Be able to carry out basic statistical analyses. | 91.3 | L7. Support formulation of job descriptions. | 90.7 | L7. Support formulation of job descriptions. | 95.9 |
| K7. Present investigation and results in the format of a research based report. | 101.3 | K7. Present investigation and results in the format of a research based report. | 97.4 | K6. Be able to carry out basic statistical analyses. | 98.5 |
